# Proteomic approaches to the characterization of protein thiol modification

**DOI:** 10.1016/j.cbpa.2010.11.003

**Published:** 2011-02

**Authors:** Edward T Chouchani, Andrew M James, Ian M Fearnley, Kathryn S Lilley, Michael P Murphy

**Affiliations:** 1 MRC Mitochondrial Biology Unit, Hills Road, Cambridge CB2 0XY, United Kingdom; 2 Department of Biochemistry, Cambridge System Biology Centre, University of Cambridge, Cambridge CB2 1GA, United Kingdom

## Abstract

Protein cysteine residues are central to redox signaling and to protection against oxidative damage through their interactions with reactive oxygen and nitrogen species, and electrophiles. Although there is considerable evidence for a functional role for cysteine modifications, the identity and physiological significance of most protein thiol alterations are unknown. One way to identify candidate proteins involved in these processes is to utilize the proteomic methodologies that have been developed in recent years for the identification of proteins that undergo cysteine modification in response to redox signals or oxidative damage. These tools have proven effective in uncovering novel protein targets of redox modification and are important first steps that allow for a better understanding of how reactive molecules may contribute to signaling and damage. Here, we discuss a number of these approaches and their application to the identification of a variety of cysteine-centered redox modifications.

## Introduction

**Figure 1:**
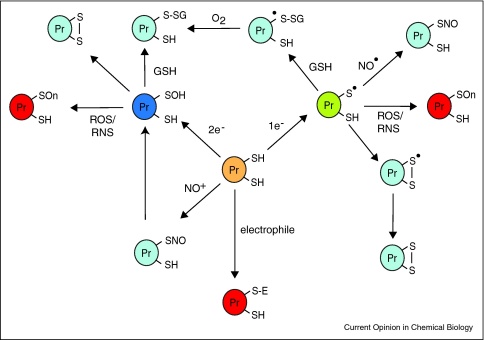
Potential cysteine modifications that may contribute to protein redox regulation and protection against damage. Proteins containing sensitive surface thiols (orange) can be oxidized by one electron (green) or two electron (dark blue) pathways. Generation of a thiyl radical by one electron oxidation can lead to subsequent glutathionylation by reduced gluathione, *S-*nitrosation by nitric oxide, or the formation of an intraprotein disulfide. Two electron oxidation can lead to the formation of a sulfenic acid, which subsequently can become further modified by glutathionylation or the formation of an intraprotein or interprotein disulfide. Some protein thiols can also become *S*-nitrosated by transnitrosation by NO containing species such as *S*-nitrosoglutathione. In contrast to these reversible modifications, protein thiols can become irreversibly altered (red) by prolonged exposure to ROS and RNS or by reaction with electrophiles. Methods for the detection of reversible and irreversible modifications to protein thiols are discussed in the text.

Modification of cysteine residues by reactive oxygen species (ROS), reactive nitrogen species (RNS) and electrophiles has emerged as a significant means of altering the structure and function of many proteins [1–6]. Reversible oxidation of certain protein thiol groups plays key signaling roles in a range of physiological processes, for example in the regulation of tyrosine phosphatase activity [7], the redox regulation of transcription factors [8] and in T cell activation during the immune response [9]. The reactivity of protein thiols with ROS, RNS and electrophiles additionally underlies their important role in defense against oxidative damage and xenobiotics [1–5,10]. In all of these processes there are a broad range of reactions that can occur to the cysteine thiol (Figure 1). Whether a modification occurs depends on a number of factors including the local environment of the cysteine residue, its proximity to the relevant reactive species, its p*K*_a_, solvent exposure and subcellular location [1,6,11,12^••^]. Additionally, some of these cysteine modifications are reversible by the action of reductive processes through the thioredoxin and glutathione systems [13,14]. Reversible thiol modifications include glutathionylation [15], mixed disulfide formation with low molecular weight thiols, sulfenic acid formation [3], *S*-nitrosation (*S*-nitrosylation) [16], *S*-acylation [17], sulfenylamide formation [18], and the generation of intraprotein and interprotein disulfides [19,20]. In addition to reversible modifications, there are a number of cysteine adducts that can form irreversibly due to reactions with electrophiles, which generally produce thioether products [10]. Similarly, the prolonged exposure of cysteine residues to ROS and RNS can also lead to the formation of irreversibly modified forms, such as sulfinic or sulfonic acids [21,22].

**Figure 2:**
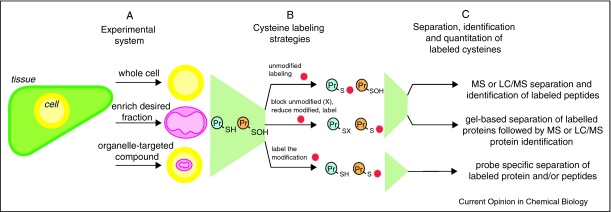
General strategies for the identification of redox active cysteine residues. **(a)** The proteome can be assessed from the entire cell, or a particular subcellular fraction enriched before cysteine labeling. Alternatively, organelle-targeted compounds can be used to elicit an organelle specific effect. **(b)** Three general strategies are employed for the labeling of redox active cysteines (orange protein thiol): (Top) Unmodified cysteine residues are labeled with a detectable probe (red probe) while modified cysteines are not labeled. The decrease in the extent of labeling indicates the extent of modification. (Middle) To label reversibly modified cysteine residues, all unmodified cysteines are first blocked by reaction with a thiol reagent such as NEM. Then reversibly modified cysteines are selectively reduced and labeled with a detectable probe (red probe). (Bottom) To label a particular type of cysteine modification, such as a sulfenic acid, a chemoselective probe that reacts only with the modified cysteine is used (red probe). **(c)** Subsequent separation and identification of the proteins containing selectively labeled cysteine residues. (Top) LC/MS or LC/MS/MS methods to separate and identify labeled peptides. (Middle) Gel-based separation of proteins, often followed by LC/MS or LC/MS/MS methods to separate and identify labeled peptides. (Bottom) Methods to detect a chemo-specific probe.

These protein modifications may contribute to oxidative damage, to the defense against oxidative stress and xenobiotics, or be part of redox signaling pathways. Consequently, it is of interest to be able to identify both the proteins and the cysteine residues affected, to determine the nature of the modification to the cysteine residue and to quantify the extent of the modification occurring during pathology or redox signaling. The development of thiol-reactive probes for the labeling of cysteine residues and proteomic approaches for the separation and identification of proteins containing these residues has allowed for the identification of novel protein targets. One subset of protein thiols that may be of particular interest are those in mitochondria, as these thiols are most likely to be involved in antioxidant defense against ROS production by the mitochondrial respiratory chain as well as in redox signaling. Additionally, the protein thiol content in mitochondria is high and the high local pH (∼8) makes surface thiols within this compartment more reactive [23]. Generally the study of mitochondrial protein thiols is conducted using isolated mitochondria; however, the use of mitochondria targeted compounds, such as MitoSNO [24] and (4-iodobutyl)triphenylphosphonium [25,26] enable the selective modification of mitochondrial protein thiols within more complex systems, such as cells and whole organisms. Most of the approaches used for the study of mitochondrial protein thiols can be applied to the investigation of other sub-cellular compartments or of the entire cell (Figure 2a).

Here we discuss the general methods available for the labeling of protein thiol modifications by selective probes and the separation and identification of the proteins containing particular cysteine redox modifications. In all cases the strategies are given in general terms and readers are referred to references for technical details from representative studies. When discussing these methods an effort has been made to mention techniques used to identify endogenously produced modifications or *in vivo* redox status because these approaches tend to be the most sensitive and relevant for wider application.

## General strategies to screen for protein thiol modifications

Many thiol modifications on cysteine residues are relatively labile and thiols themselves are prone to artifactual modification during protein isolation and labeling. Therefore an essential prerequisite for reliable screening for protein thiol modifications in biological samples is the efficient trapping of the native redox state of the thiol proteome [27]. This is generally done using a reactive thiol alkylating reagent such as N-ethyl maleimide (NEM) to block all free thiols, a step which is sometimes preceded by treatment with strong acid to protonate the thiols and render them less reactive [27]. There are three general approaches that are used for the labeling of cysteine residues within samples for most redox proteomic studies (Figure 2b). Either unmodified protein thiols are alkylated with a thiol specific probe that contains a reporting group that enables the labeled thiols to be detected [28–30]. Then loss of this signal is assessed as an indication of protein thiol modification (top). Alternatively, unmodified protein thiols are blocked with an unlabeled alkylating reagent, often NEM, and then reversibly modified protein thiols are selectively reduced and labeled by reaction with a detectable thiol probe (middle) [31^••^,32^••^]. Finally, the thiol modification of interest is labeled by reaction with a specific probe that is designed to react only with the modified protein thiol and render it detectable directly (bottom) [33^•^,34]. Following one of these preparations, the selectively labeled protein thiols can be assessed by a range of analytical procedures based on the nature of the thiol label that has been employed (Figure 2c). Typically, the labels attached to the cysteine residues are biotinylated, fluorescently conjugated, or isotopically modified derivatives of the thiol alkylating reagents NEM or iodoacetaminde (IAM). Such labeling procedures allow for separation of the proteins by gel electrophoresis followed by the identification of the labeled protein by peptide mass fingerprinting, or by the separation and identification of the labeled peptides by liquid chromatography–mass spectrometry (LC–MS). The details of these labeling methods are first discussed below, followed later by a comparison of the various types of thiol-reactive probe molecule that can be used and procedures that can be employed to separate and identify the labeled proteins and peptides.

## Measuring loss of selective labeling due to thiol modification

**Figure 3:**
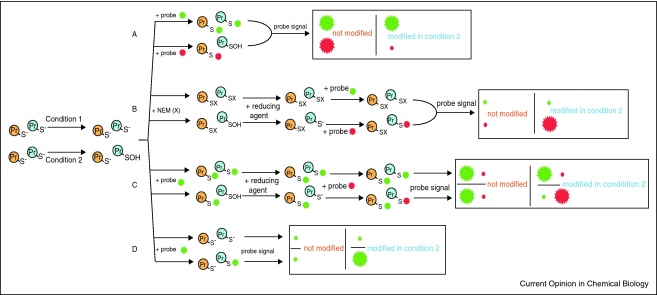
Strategies for the labeling and identification of proteins containing redox sensitive cysteines. Control samples (condition 1) are compared to samples subjected to conditions that modify certain protein thiols (condition 2). In this case a sulfenic acid modification is shown. **(a)** Identification by loss of labeling due to thiol modification. Unmodified protein thiols are labeled with different probes in each condition. Comparison of each probe signal from a combined sample determines if a thiol remains unmodified (equal signal) or is modified in one condition (diminished signal). **(b)** Identification of reversibly modified cysteine residues using a sample mixing approach appropriate for DIGE. Unmodified thiols are first blocked with a non-detectable alkylating reagent such as NEM (X). A reducing agent is applied to reduce all reversible modification, or to selectively reduce a particular modification of interest. Newly reduced thiols are then labeled with different probes in each condition. Mixed samples will show a difference in labeling of reversibly modified thiol proteins. **(c)** Identification of reversibly modified cysteine residues using a sample mixing approach appropriate for LC/MS. Unmodified thiols are blocked with a probe (green). A reducing agent is applied to reduce any reversible modification or a particular modification of interest. Newly reduced thiols are then labeled with a second probe (red). Samples from individual conditions are resolved by LC/MS, so the signal ratio of probes for a particular cysteine indicates the extent of the modification being studied. **(d)** Identification of redox sensitive cysteines by chemoselective probes for a particular modification. A chemoselective probe for a modification of interest alkylates modified cysteine residues (green). Samples are resolved depending on the nature of the probe used and the presence of a signal is indicative of a modification.

A simple but limited strategy for the identification of modified protein thiols is to label all unmodified protein thiols with detectable thiol-reactive probes (Figure 2b, top) [28–30]. Then control labeled protein samples can be separated by electrophoresis, or derived peptides are separated by LC–MS, and compared with related samples prepared under stressed or oxidant-treated conditions. Probe signal loss between conditions is indicative of both reversibly and irreversibly modified protein thiols (Figure 3a). However, the reliance on measuring signal loss, instead of signal increase over baseline, is a significant limitation to the sensitivity of this approach since most intracellular protein thiols are maintained in a reduced state by the glutathione and thioredoxin systems. Consequently, this strategy is best suited for determining changes due to high concentrations of oxidants or in simple protein samples. The signal loss method can in principle be adapted to detect only irreversible protein thiol modifications by the treatment of samples with a thiol reductant, such as *tris*(2-carboxyethyl)phosphine (TCEP) or dithiothreitol (DTT) before labeling. In this case any signal loss would be attributable to irreversibly oxidized thiols.

## Selective reduction of reversible protein thiol modifications

A more widely used strategy, and one that is generally the most useful for the detection of reversibly modified protein thiols, blocks all unmodified thiols with a general thiol reagent such as NEM. This is followed by the selective reduction and labeling of all reversibly modified cysteine residues with a thiol probe. All redox-sensitive cysteine residues will be labeled and screened for by this procedure, regardless of the nature of the reversible modification (Figure 3b and c). This is advantageous when the conditions being compared involve a range of reversible modifications, of which the combination and proportion are unknown. Although this method casts the widest net, it does not allow for the identification of a modification for a particular protein thiol of interest, which requires a selective approach. One example of this approach was in seeking to identify mitochondrial thiol proteins sensitive to low levels of endogenous ROS production [31^••^,35]. For this, mitochondria were treated as described in Figure 3b such that unmodified thiols were blocked with NEM and reversibly modified residues were reduced using DTT and subsequently labeled using a fluorescently labeled thiol probe [31^••^,35]. Using a slightly different strategy (Figure 3c) Leichert *et al.* were able to identify a number of protein thiols in *Escherichia coli* sensitive to exogenous hydrogen peroxide (H_2_O_2_) and hypochlorite using TCEP as a thiol-specific reductant [32^••^]. This strategy differs in that the initial blocking of exposed thiol was done with a thiol-specific probe instead of NEM, and the labeling of oxidized protein thiols after reduction with TCEP was done using an isotopically labeled thiol probe, so that the ratio of unmodified to modified cysteine residues could be assessed.

The above methods lead to the labeling of all reversible cysteine modifications and are powerful means of screening for all protein thiols sensitive to modification in a particular biological condition. However, there is also considerable interest in differentiating between different types of reversible cysteine modifications. The *S*-nitrosation of protein thiols is one such important modification. The strategy for identification of *S*-nitrosated protein thiols on a proteomic scale involves the selective reduction of protein *S*-nitrosothiols using either ascorbate or the combination of ascorbate and copper (II) [36–39,40^•^,41]. Highlighting the potential to determine cysteine targets *in vivo* using ascorbate reduction conditions, Sun *et al*. were able to identify a number of *S-*nitrosated proteins generated endogenously in ischemic preconditioned and *S*-nitrosoglutathione treated rat hearts [38]. However, recent studies on the selectivity of ascorbate as a protein *S-*nitrosothiol reductant suggest that at low concentrations it is insufficient and at high concentrations it is non-specific [42^•^,43,44]. So, on a proteomic scale where sensitivity and selectivity are of utmost importance, the Hogg group has demonstrated that the selective reduction of *S*-nitrosated proteins is best accomplished using a combination of ascorbate at low concentrations and copper (II) [39,42^•^]. Using ascorbate and copper (II) in combination generates copper (I) which reacts in a highly selective fashion with *S*-nitrosothiols while leaving other thiol modifications unaffected [39,42^•^,45]. These improved conditions for selective reduction have since been successfully used for sensitive detection of *S-*nitrosated proteins in cells as well as mitochondria [39,40^•^].

Disulfide formation as a consequence of cysteine oxidation is a prevalent thiol modification. Proteomic strategies have been developed for the identification of both intraprotein and interprotein disulfides. Vicinal dithiols, which are likely to form intraprotein disulfides because of their proximity, can be identified on the basis of a selective labeling and reduction strategy. Protein dithiols in reduced protein samples can be selectively blocked with the dithiol specific reagent phenylarsine oxide (PAO) and then all other thiols alkylated with NEM. Subsequently, PAO-blocked dithiols are selectively reduced using the PAO-specific reducing agent 2,3-dimercaptopropanesulfonic acid (DMPS) and labeled with an alkylating probe [19,46,47]. Identification of novel proteins that undergo inter-protein disulfide formation is also possible using diagonal electrophoresis [48]. Protein samples are first resolved by non-reducing SDS-PAGE so that all thiol modifications remain intact. Then samples are resolved in the second dimension with DTT incorporated into the running medium. By incorporating the reduction step at this point, proteins involved in inter-protein disulfide linkages will migrate off the diagonal and can be subsequently identified by peptide mass fingerprinting or with an antibody on a western blot if candidate proteins are suspected. The reliance of this technique on electrophoresis limits the potential resolving power for complex protein mixtures. This lack of sensitivity can be addressed to some extent if a thiol specific fluorescent probe is incorporated during the reduction step. Although this would focus on the cysteine residues, in this case other thiol modifications in addition to inter-protein disulfides would also be labeled.

As both the glutathione and thioredoxin systems are critical for the maintenance of protein thiol redox homeostasis, techniques have been developed to identify the protein targets of these interactions. Lind *et al.* used a mutant glutaredoxin from *E. coli* to selectively reduce glutathionylated proteins following the general scheme described in Figure 3b [49^•^]. Although this strategy may identify constitutively glutathionylated proteins it is unclear if the mutant glutaredoxin is capable of reducing all glutathionylated proteins. Sensitive strategies for the identification of thioredoxin-conjugated proteins have relied on the blocking of unmodified thiols, followed by the treatment of oxidized thiols ± thioredoxin and blocking of thioredoxin-reduced thiols. Finally, oxidized thiols not affected by thioredoxin treatment are reduced and labeled resulting in a signal [50^•^]. Decreased signal probe intensity in thioredoxin treated samples is indicative of a target cysteine residue. Recently, Benhar and colleagues used a combined strategy of selective reduction of protein *S*-nitrosothiols and thioredoxin conjugation to specifically determine *S*-nitrosated targets of thioredoxin action [51]. Using stable isotope labeling by amino acids in cell culture (SILAC), entire proteomes can be differentially labeled with light or heavy lysine. Subsequently, protein samples are isolated and treated with an *S-*nitrosating agent, with one sample being exposed to exogenous thioredoxin and thioredoxin reductase. Both samples are subjected to reduction of protein *S*-nitrosothiols as described above and labeled. By comparing probe signals between samples, *S-*nitrosated thiol signals that are diminished in the thioredoxin-treated samples can be identified.

## Selective reaction of particular protein thiol modifications

Although some redox proteomic methodologies make use of specific reduction of the cysteine modification of interest, others employ probes that react specifically with a particular modification thereby circumventing the requirement for a reduction step. These methods and the modifications they are applied to are outlined below and the general approach is described in Figure 3d. In general, this strategy is advantageous because the methods allow for labeling within the system, affording a low chance of redox homeostasis disruption and artifactual labeling. However, since quantification with respect to the proportion of modified to unmodified cysteine cannot be made, these methods can only determine the presence of a modification.

A number of proteomic strategies have been developed for the identification of sulfenic acids using chemoselective probes based on derivatives of 5,5-dimethyl-1,3-cyclohexadione (dimedone). Conjugation of the sulfenic acid-specific dimedone to fluorophores or biotin has allowed for proteomic screens of these conjugates [33^•^,52,53]. More recently, Leonard *et al.* developed a membrane permeable propyl azide derivative of dimedone capable of labeling sulfenic acids in cells while allowing for downstream selective coupling with an alkyne or phosphine biotin tag [12^••^]. This strategy foregoes the requirement for reduction of sulfenic acids and avoids potential disruption of redox homeostasis since tagging can occur within intact cells.

An alternative strategy for the identification of glutathionylated proteins is based on metabolic labeling. Fratelli *et al.* metabolically labeled the glutathione pool of T-cells using [^35^S]-cysteine under a variety conditions applying exogenous oxidative stress [34]. Treatment with [^35^S]-labeled cysteine in conjunction with the protein synthesis inhibitor cycloheximide allowed for the majority of the labeled cysteines to be incorporated into the glutathione pool. Then [^35^S]-glutathionylated proteins were separated by two-dimensional electrophoresis and assessed by radiofluorography. Among the limitations of this approach are that proteins glutathionylated before metabolic labeling will not be detected. In addition the sensitivity of the radiofluorography system for detecting subtle changes is less robust when compared to fluorescent or MS probes that enable control and modified samples to be compared more directly.

## Separation and identification of proteins containing selectively labeled thiols

Choosing from the number of approaches available for the selective labeling of protein thiol modifications is the first step in any redox proteomics study, and these strategies have been summarized in Table 1. Consideration must be paid to the subsequent separation and identification of the proteins containing the labeled thiols. The approaches to do this rely on electrophoresis, LC–MS and mass spectrometry, either alone or in combination, and the advantages and disadvantages of the various approaches are discussed below.

Gel based protein separation, typically by the two-dimensional electrophoresis (2DE) of complex protein samples, has been used broadly to separate many labeled thiol proteins. Essential to obtaining reliable results using this approach is an experimental design that minimizes variability between the samples being compared, otherwise false positive and false negative rates will be high. Since a significant source of variability in 2DE is inter-gel variation when comparing gel pairs, the difference in gel electrophoresis (DIGE) method has been developed because it allows for comparison of two samples within the same gel [54]. DIGE makes use of fluorescently resolvable thiol alkylating probes that allows multiple samples to be combined and compared on the same gel. By combining protein samples with modified thiols alkylated with these probes, differences in fluorescence can be compared on the same gel and the presence of a modification reliably established using the labeling strategy outlined in Figure 3b [35]. Other sources of variability include biological variability between biological replicates and technical variability in sample workup before sample mixing [55]. One way in which these forms of variability can be minimized is by the application of sample pooling based on biological variance analysis (BVA), which has shown to be an effective means of minimizing false positive and false negative results [40^•^,55,56]. These considerations are particularly important for studies where the thiol modification may affect only a small proportion of the protein thiols present (e.g. low levels of endogenous ROS production or protein *S*-nitrosation) and high statistical power is desired.

Although gel based methods allow for the identification of thiol proteins sensitive to redox modifications, the modified cysteine(s) on the protein and the extent of the modification cannot be obtained. In addition, the use of 2DE results in the underrepresentation of hydrophobic membrane proteins because of their relative incompatibility with the essential isoelectric focusing step. Furthermore, all gel-based methods tend to favor the identification of abundant proteins. Alternative means of gel-based separation can be applied to these proteomic screens; for example blue native-PAGE separation of mitochondrial respiratory complexes [57].

Using thiol alkylating probes amenable to LC–MS based separation affords the potential for significantly more information to be obtained from a redox proteomic study. To differentiate between modified and unmodified thiols in this way, isotopically light and heavy thiol alkylating probes are used to tag unmodified and modified thiol groups, respectively (Figure 3c). Following digestion of all proteins with a peptidase such as trypsin, cysteine containing peptides are separated and identified by LC–MS and those containing modified thiols will appear as peak pairs corresponding to the isotopically light labeled thiol (unmodified) and the isotopically heavy labeled thiol (modified), separated by the mass difference between the probes. There are a number of advantages to this approach over gel-based methods. In addition to identification of the thiol protein sensitive to a particular modification, the sensitive cysteine residue(s) can be determined. Furthermore, the use of two probes on an individual sample for analysis by LC–MS allows for internal comparison and can give a reliable measurement of the ratio of unmodified to modified cysteine. However, unlike gel-based methods where the background due to non-cysteine and unlabelled cysteine containing proteins is not an issue, the LC–MS analysis of complex samples would contain a significant background from these irrelevant sources. For this reason a labeling method using isotope coded affinity tag (ICAT) technology, which allows for affinity-purification of ICAT labeled peptides before their separation and identification by LC–MS is often a more robust approach [32^••^]. A recent extension of this approach is the development of cysteine tandem mass tags (cysTMTs) which allow for the selective isolation of modified cysteine peptides, as is the case in ICAT, as well as the potential for more accurate quantification by LC/MS/MS and the comparison of up to 6 conditions within a single experiment (http://www.piercenet.com). Although these methods greatly increase sensitivity by concentrating the selectively labeled peptide only, it is likely that affinity purification selects for the most abundant cysteine containing peptides and low abundance proteins could be left undetected.

## Conclusion

Many redox-active cysteine residues play central and varied roles in redox signaling pathways and in the control of redox homeostasis and the response to oxidative stress and xenobiotics. To identify these cysteines and determine the functional significance of their modifications, a number of sensitive redox proteomic strategies have been developed. Using these approaches it is possible to identify those proteins that contain cysteine residues that are modified. In some cases it is also possible to determine the nature of the modification or at least indicate if the modification is reversible or irreversible, and perhaps identify the cysteine residue of interest. The use of LC/MS or LC/MS/MS can then enable the extent of the modification to be determined. However, as with all proteomic approaches, the methods outlined here only give a first indication that a particular condition affects thiols on a particular protein and perhaps a certain cysteine residue. In most cases this will be the first step in determining the physiological and pathological significance of the modification and subsequent orthogonal techniques will be essential to determine the extent of the modification, a critical factor in determining its significance, and the effect of a cysteine modification.

## References and recommended reading

Papers of particular interest, published within the period of review, have been highlighted as:

• of special interest
•• of outstanding interest

## Figures and Tables

**Table 1:** A summary of the redox-proteomic strategies available for the identification of particular protein thiol modifications. Strategies are organized based on the type of modification each screen will identify. A brief outline of the labeling strategy for each protocol is included along with helpful references to representative studies that make use of each technique

Cysteine modification	Labeling strategy	Helpful studies that use various methods of resolution	References
Reversible and irreversible	Label unmodified thiols—Figure 3a	MS	[28]
MS	[29]
MS and DIGE	[30]

Reversible	Sample mixing strategy—Figure 3b	DIGE	[31^••^]
(1) Block unmodified thiols
(2) Reduce modified thiols with DTT or TCEP
(3) Label nascent thiols

Reversible	Differential alkylation strategy—Figure 3c	MS	[32^••^]
(1) Label unmodified thiols
(2) Reduce modified thiols with DTT or TCEP
(3) Label nascent thiols

Protein *S*-nitrosothiols	(1) Apply sample mixing or	DIGE–ascorbate/copper	[39,40^•^]
Differential alkylation strategy	DIGE–ascorbate	[38]
(2) Reduce *S*-nitrosated thiols with Cu(II) and ascorbate	IP–ascorbate	[36]
(3) Label nascent thiols	IP–ascorbate	[37]
	MS–ascorbate	[41]

Protein dithiols	Sample mixing strategy–Figure 3b	DIGE	[47]
(1) Label dithiols with PAO
(2) Block unmodified thiols
(3) Reduce PAO-modified thiols with DMPS
(4) Label nascent dithiols

Inter-protein disulfides	(1) Block unmodified thiols	Diagonal-PAGE	[48]
(2) Resolve by nonreducing PAGE
(3) Reduce with DTT or TCEP
(4) Resolve by diagonal reducing PAGE

Sulfenic acids	Label directly—Figure 3d	MS, gel	[33^•^]
(1) Label sulfenic acids with dimedone-based probes	IP, MS	[53]
	IP, MS	[12^••^]

Glutathionylation	Metabolic labeling—Figure 3d	Gel	[34]
(1) Incubate samples with ^35^S-cys so newly glutathionylated proteins are metabolically labeled

Glutathionylation	(1) Apply sample mixing or differential alkylation strategy	Gel	[49^•^]
(2) Reduce with mutant *E. coli* glutaredoxin
(3) Label previously glutathionylated thiols

Thioredoxin target	(1) Apply sample mixing strategy	MS	[50^•^]
(2) Block unmodified thiols
(3) Incubate ± thioredoxin
(4) Block newly reduced thiols
(5) Reduce with DTT or TCEP
(6) Label thiols

Protein *S*-nitrosothiol targets of thioredoxin	(1) SILAC metabolic labeling	MS	[51]
(2) Treat with nitric oxide donor
(3) Incubate ± thioredoxin
(4) Block unmodified thiols
(5) Protein *S*-nitrosothiol reduction
(6) Label *S*-nitrosated thiols
